# Comments on “Rolling epidemic of Legionnaires’ disease outbreaks in small geographic areas”

**DOI:** 10.1038/s41426-018-0152-8

**Published:** 2018-09-05

**Authors:** Sharon K. Greene, Robert Fitzhenry, Bruce Gutelius

**Affiliations:** Bureau of Communicable Disease, New York City Department of Health and Mental Hygiene, Long Island City, NY 11101 USA

In their recent article, MacIntyre et al.^1^ review a series of Legionnaires’ disease (LD) outbreaks in New York City (NYC) and Sydney using information collected from public sources including news reports—a highly biased sample because of limited public information related to LD outbreaks in other jurisdictions. This article has critical problems with conception, reported data, and results interpretation. We believe it is important to inform your readers, and the community of researchers and practitioners working to reduce LD incidence, of these issues.

The authors claim that the degree of geographic and temporal clustering of LD outbreaks in NYC and Sydney is “unusual” as compared with other jurisdictions. However, the authors did not consider detection bias and underlying population characteristics of cities as likely alternative explanations. LD outbreaks in NYC are likely more completely detected and reported than in other jurisdictions because of complete and timely case notification via electronic laboratory reporting, automated cluster detection processes^2–4^, and adequate resources to investigate and report when cases are suspected to have a shared exposure. The authors did not provide evidence that the degree of clustering in these cities is statistically unlikely to occur by chance alone, accounting for this detection bias. In addition, large cities—by definition, small geographic areas—have large numbers of people with underlying conditions that can increase the risk of LD. It is thus expected that LD outbreaks would be more likely to occur and be detected in cities with large at-risk populations than in rural areas.

The use of phrases such as “contamination of potable water” and “potable water supply is a more common source in US outbreaks” is inaccurate when referring to LD cases occurring in NYC. This phrasing implies that outbreaks in NYC have been linked to the municipal water distribution system, which is incorrect. For buildings in NYC where more than one case has occurred among tenants within 12 months, water testing has sometimes shown evidence of amplification of *Legionella* within complex building water systems (i.e. within the building), with no linkage to the municipal water distribution system. Similarly, the terms “rolling epidemic” (used only once, in the title) and “rolling outbreak” strongly imply that the outbreaks were linked, but the authors present no evidence this is the case.

Figure 4 and related text conflate data from New York State with data from New York City. The Figure 4 caption indicates that these are New York State data, while the text referring to Figure 4 indicates the data are from New York City (“*A total of 32 outbreaks were reported in NYC from July 2003 to November 2012, and of these, only 5 (16%) were due to cooling towers. Figure 4 shows the source of these different outbreaks, with most being caused by contamination of potable water*.”). If the Figure 4 caption is accurate, then these results are clearly misrepresented in the text, and the implications of these data are quite different, as New York State, including New York City, has a total population of ~20 million people and includes 62 counties over ~55,000 square miles, with thousands of public water systems; New York City, by itself, has a total population of ~8.6 million people, includes 5 counties over ~304 square miles, and has one municipal water system. To clarify, the authors should have provided a linelist for each outbreak in Figure 4, including variables such as data source, outbreak timing, number of persons affected, and outbreak source.

Furthermore, the article contains self-contradictory language. As one example, in the discussion, climate change is said to be a long-term effect and have little impact on LD cases, but this is followed by stating that “slightly warmer temperatures” support rolling outbreaks due to unseasonably warm weather. The authors have not performed the necessary data collection or analyses to support such conjecture. As another example, the results for outbreak 2 in Morris Park state that “the strain found in the cooling tower at the Bronx Psychiatric Centre matched samples taken from four patients.” However, the discussion asserts the opposite, i.e., that “no data have been reported on matching to date on the remaining outbreaks” other than the Opera House Hotel outbreak. The latter is incorrect as matching data were provided by Lapierre et al., which showed that clinical strains obtained from outbreak 2 in Morris Park did match by whole genome sequencing.^5^ It should be noted that the strains identified as the source in outbreak 1 (South Bronx) and outbreak 2 (Morris Park) are not identical strains.^5^ Thus, these outbreaks are not linked by epidemiological or laboratory data and represent separate events.

Finally, the authors provide no analysis or citations to support their assertions that: “in relation to the Bronx, the Opera House Hotel and Morris Park outbreaks, it is unusual for so many cooling towers to be contaminated at one time” and “the reporting standards are generally good in both countries.” Key references presented to support points of discussion are not peer-reviewed while relevant peer-reviewed literature^4,5^ is uncited.

Health department practitioners who originally collect, analyze, and report on communicable disease data are well-positioned to provide meaningful local context and interpretation. We strongly encourage researchers to reach out to health department practitioners when conceiving articles using such data, and journal editors to invite such practitioners to serve as peer reviewers for such articles prior to publication
